# Radiation therapy for carcinoma of the uterine cervix: comparison of two brachytherapy schedules

**DOI:** 10.1093/jrr/rrt226

**Published:** 2014-02-20

**Authors:** Masashi Chatani, Kazuki Tsuboi, Masayuki Yagi, Kanta Fujiwara, Rika Tachimoto

**Affiliations:** Department of Radiation Oncology, Osaka Rosai Hospital, 1179-3 Nagasone-cyo, kita-ku, Sakai, Osaka, Japan

**Keywords:** carcinoma, uterine cervix, RALS, high dose rate

## Abstract

We compared the survival rates and late effects for two groups of cervical cancer patients treated with almost the same external radiotherapy but different remote afterloading systems (RALS) for high-dose-rate intracavitary radiation therapy regimens. A total of 218 patients with carcinoma of the uterine cervix were treated. For 98 patients, intracavitary brachytherapy was delivered with 6–7.5 Gy/fraction to Point A (Group A), and for 120, 5 Gy/fraction with a modified source step size (Group B). The 3-year cause-specific survival rates by stage and treatment schedule were Group A: 91% and Group B: 96% in Stage I, 89% and 92% in Stage II, 64% and 75% in Stage III, 44% and 69% in Stage IV. The survival curves did not reveal any statistically significant differences at any stage. The 3-year cumulative local failure rates were 14% in Group A and 7% in Group B (*P* = 0.1202), while the actuarial rates of developing rectal complication (Grade 2 or more) at 3 years were 25% in Group A and 4% in Group B (*P* < 0.0001). This retrospective analysis suggests that a low dose per fraction with modified source step size is advantageous because of yielding almost the same local control but with fewer rectal complications.

## INTRODUCTION

In Japan, remote afterloading system (RALS) with high-dose rate intracavitary therapy has been recognized as an effective and safe treatment modality for carcinoma of the uterine cervix over the past few decades. In 1991, we reported a prospective randomized study concerning the Point A dose (7.5 Gy vs 6 Gy) by Co-RALS and showed no statistically significant difference between the two treatment schedules with respect to survival rates [1]. In 1998, Ir-RALS was commenced, but rectal complications were relatively frequent, in spite of the same Co-RALS regime being employed. In February 2004, the Ir-RALS dose was reduced to 5 Gy/fraction with a modified source step size. This study shows the results of two treatment regimens of the Ir-RALS.

## MATERIALS AND METHODS

This retrospective study was approved by the Institutional Review Board of Osaka Rosai Hospital, and all patients provided informed written consent.

### Patients and treatment schedules

From June 1998 through December 2009, a total of 218 patients with carcinoma of the uterine cervix were treated with curative intent using external beam radiotherapy (EBRT) and RALS. Clinical staging was made according to the staging system of the UICC (1987) without general anesthesia by a gynecologist and a radiation oncologist. Other investigations, such as cystoscopy, proctoscopy, abdominal and pelvic computed tomography or magnetic resonance imaging were routinely performed, and rectosigmoidoscopy was used for advanced cases (T3 and T4). Between June 1998 and January 2004, 98 patients received EBRT with RALS at a dose of 6–7.5 Gy/fraction. An intermediary analysis in January 2004 indicated that the incidence of rectal complication was relatively high, and from February 2004, the RALS dose was reduced to 5 Gy/fraction with a modified source step size [2]. Thus, two groups of patients with different treatment schedules were included in the present study (Table 1). A total of 98 patients were included from the first period (Group A) and 120 patients from the second period (Group B). Treatment schedules and the patient distribution are shown in Tables 2 and 3. There were significant differences in age distribution, applicators and chemotherapy between Groups A and B.

**Table 1.** Treatment schedule

Arm A	External RT (Gy)		Ir-RALS (Gy/fraction)
	Whole pelvis	Center shield	
IB–IIA	14	28	36/6–37.5/5
IIB–IIIB	28	18–28	30/4–5
IV	40	20	24/4–22.5/3
Arm B	External RT (Gy)		Ir-RALS (Gy/fraction)
	Whole pelvis	Center shield	
IB–IIA	20	30	25/5
IIB–IIIB	30	20–30	25/5
IV	40	20	20/4

**Table 2.** Patient distribution

	A	B	*P*-value
Age (years old)			
<70	62	93	0.021
≥70	36	27	
Stage			
IB	14	23	
II	39	41	0.663
III	36	42	
IV	9	14	
Pathology			
Squamous cell carcinoma	87	107	0.548
Adenocarcinoma/Sarcoma	11	13

**Table 3.** Patient distribution

	A	B	*P*-value
Applicator			
F–A^a^	88	37	
Henschke	3	25	0.000 1
F–W^b^	5	58	
Cylinder	2	0	
Treatment			
RT alone	82	47	0.000 1
RT + Chemotherapy	16	73	

^a^Fletcher–Williamson Asian Pacific Applicator.

^b^Fletcher–Williamson Applicator.

### EBRT and RALS

EBRT was employed using a 10-MV X-ray machine. Antero–posterior parallel opposed portals were used with a conventional five fractions per week. The upper margin of the radiation field for the whole pelvis included the upper border of the fifth lumbar vertebra; the lower margin included the inferior border of the pubic symphysis; the lateral margin was at 2 cm lateral to the bony pelvis. The lower two-thirds of the *y*-axis of the whole-pelvis field for a 3-cm width was blocked in the central shield field so as to reduce the rectal dose. The EBRT dose was performed at 2 Gy/fraction, with five fractions weekly. The dose of EBRT and RALS was altered according to stage. The dose of RALS for early lesions was greater than for advanced lesions, whereas the dose of EBRT was the reciprocal (Table 1). RALS was started from the central shield field and performed once a week during EBRT. EBRT and RALS were allowed on the same day, with a minimal separation of 6 h. Most patients were treated with an ovoid and tandem applicator, with a standardized source configuration forming the classic pear-shaped isodoses of the Manchester system. The dose was prescribed to Point A, 2 cm superior to the vaginal sources and 2 cm lateral to the cervical canal. A MicroSelectron machine with a stepping ^192^Ir source was used, and modulation of the delivered dose by individualized treatment planning was performed. In Group A, a non-shielded applicator was preferentially used, but in Group B a tungsten shield applicator (Fletcher–Williamson applicator) was selected for 48% of the patients (Table 3). Insertion of the intracavitary apparatus was carried out with diclofenac sodium or pentazocine.

**Fig. 1.**
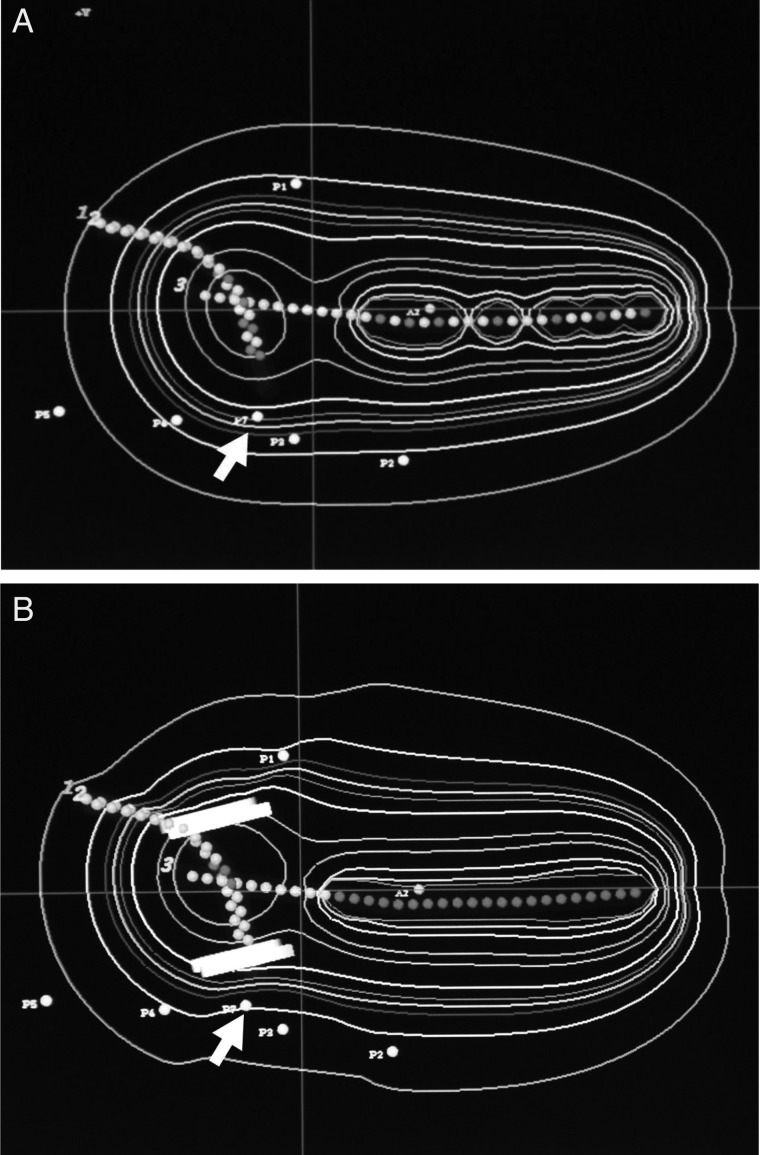
Isodose curves of Ir-RALS. The dose to the ICRU rectum point (arrow) was 138% of the Point A dose in Group A and 82% in of the Point A dose in Group B (by tungsten shielded applicator and modified source step size). From the outside, isodose curves show 50%, 80%, 100%, 110%, 120%, 140%, 200%, 150%, 400%, 600% and 800% of the Point A dose.

The source step size was also different, i.e., a 7.5–10 mm tandem source step size was used in Group A and a 2.5 mm step size in Group B; a 5 mm step × 4 points of ovoid source was used in Group A, and a 2.5 mm step ×2 points of ovoid source was used in Group B (Table 4). The dose to the ICRU rectum point was not prospectively calculated in Group A, although in Group B the dose to the ICRU rectum point was restricted to within ∼ 80% of the Point A dose by a tungsten shielded applicator, gauze packing and a modified source step size. Figure 1 shows the dose distribution in each group.

**Table 4.** Source points and weight (e.g. tandem 6 cm, small, ovoid)

Arm A	Points	Weight
Ovoid1	1•3•5•7	1
Ovoid2	1•3•5•7	1
Tandem	1••4••7•••11•••15•••19	1.14
Arm B	Points	Weight
Ovoid1	•••45•••	1
Ovoid2	•••45•••	1
Tandem	123456789101112131415161718192021	0.24/0.16

### Follow-up schedule

Patient status was followed up once a month for 1 year, then once every 3 months after radiation therapy. Duration of follow-up ranged from 3–7 years. Survival probability was calculated by the Kaplan–Meier method, and statistical significance was tested by means of a log-rank test.

## RESULTS

### Survival and local failure

**Fig. 2.**
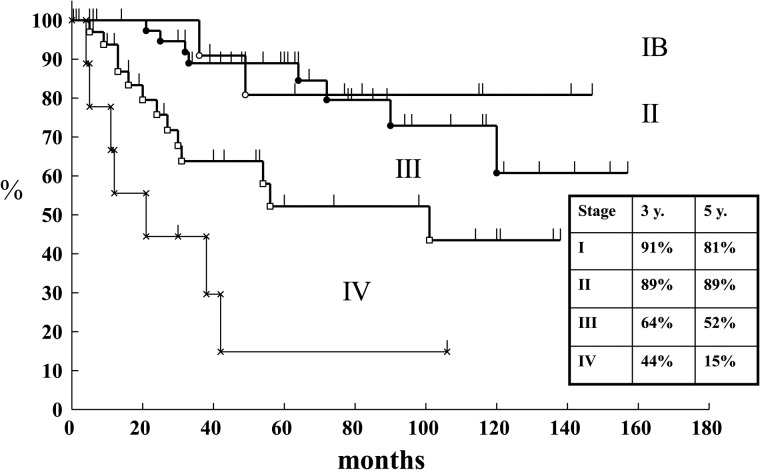
Cause-specific survival of carcinoma of the uterine cervix by stage (Arm A: June 1998–January 2004).

**Fig. 3.**
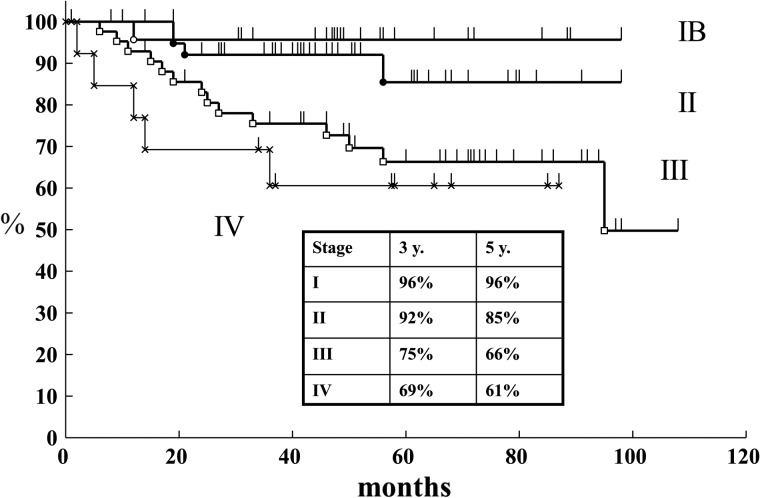
Cause-specific survival of carcinoma of the uterine cervix by stage (Arm B: February 2004–December 2009).

**Fig. 4.**
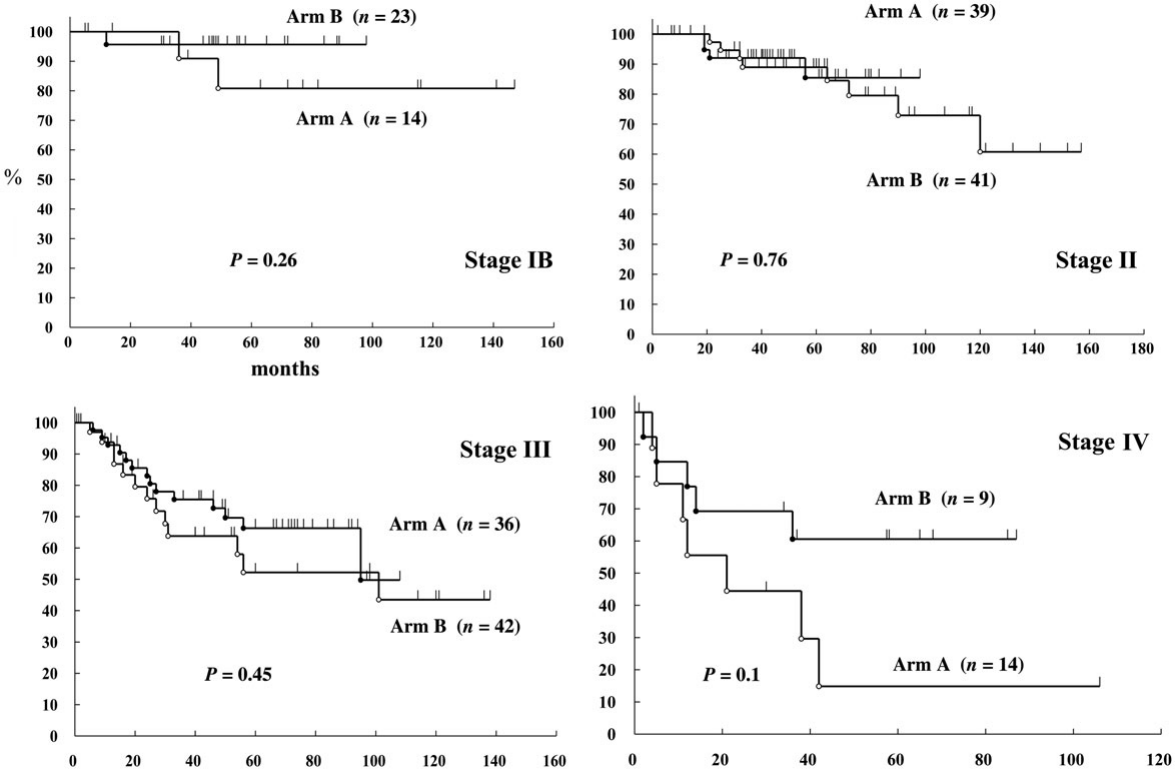
Cause-specific survival of carcinoma of the uterine cervix by treatment schedule (June 1998–December 2009).

The cause-specific survival rates by stage are shown in Figs 1 and 2. The 3-year survival rates by stage and treatment schedule were in Group A 91% and in Group B 96% in Stage I, 89% and 92% in Stage II, 64% and 75% in Stage III, and 44% and 69% in Stage IV, respectively. There was no statistically significant difference for any stage (Fig. 3). The 3-year cumulative local failure rates were 14% in Group A and 7% in Group B (*P* = 0.1202) (Fig. 4).

### Complications

**Fig. 5.**
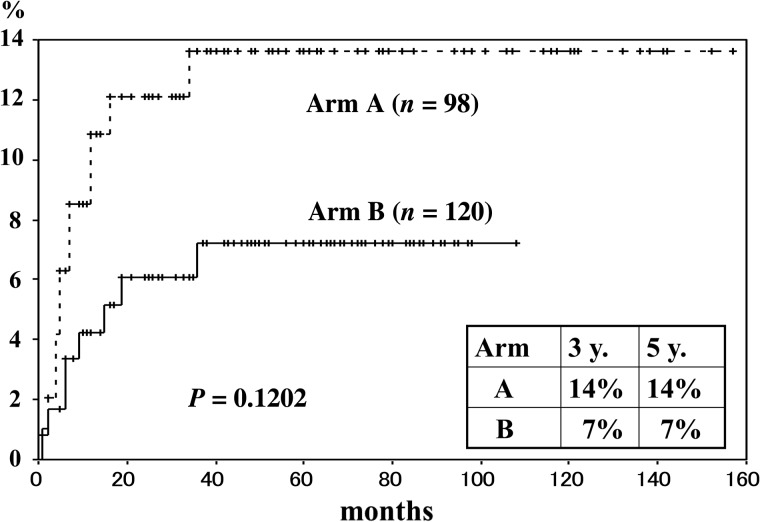
Cumulative local recurrence rate by treatment schedule (June 1998–December 2009).

The adverse events by NCI-CTCAE (ver.3) [3] are shown in Table 5, and the actuarial rate of developing rectal complication at 3 years were 25% in Group A and 4% in Group B (*P* < 0.0001), respectively (Fig. 5).

**Table 5.** Adverse events

	A (*n* = 98)	B (*n* = 120)
Rectum		
Ulcer (G2)	20	2
Multiple ulcer (G3)	0	2
Necrosis (G4)	1	0
Bladder		
Pain, hematura (G2)	2	0
Necrosis (G4)	1	0
Small intestine		
Ileus (G3)	4	0
Necrosis (G4)	1	0

By NCI-CTCAE Ver. 3.0 [3].

**Fig. 6.**
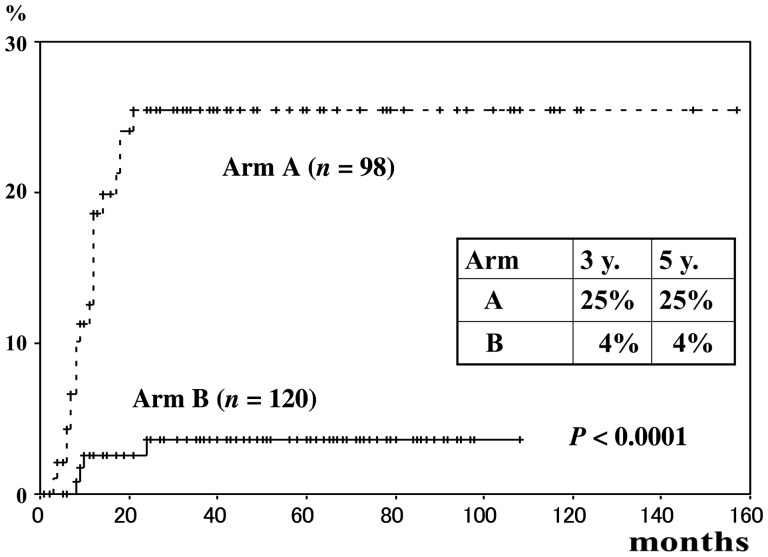
Cumulative rectal complication rate by treatment schedule (June 1998–December 2009).

## DISCUSSION

Rectal complications are often a major concern when patients with cervical carcinoma are treated with a combination of EBRT and RALS. Several analyses have been performed to establish the dose constraints for reducing the incidence of such complications. However, the dose distribution from RALS is highly inhomogeneous, and several different methods of specifying the dose constraints have been published. Moreover, the risk assessment is also complicated because the dose contribution from EBRT and RALS varies considerably from study to study [4–6, 7–8].

The standard location for the rectum reference point is established by the International Commission on Radiation Units and Measurements (ICRU). Although this point does not consistently give the location of the maximal dose to the organs [4], the point dose is significant correlated with the incidence of late rectal complications, and the biological effective dose (BED) value of 100 is a threshold of rectal complication using an α/β ratio of 3 Gy [5].

We previously reported the local control and complication rates for patients with carcinoma of the uterine cervix between 1978 and 1982 [9], and we also reported a prospective randomized study concerning the Point A dose (7.5 Gy vs 6 Gy) and showed no statistically significant difference between the two treatment schedules with respect to survival rates or complication rates by Co-RALS [1]. But in the present study by Ir-RALS, although there were no statistically significant differences between Groups A and B in terms of survival rates or local control rates, there were significant difference in rectal complication rates (Fig. 4). The reason for the discrepancy could be that there are some differences in the brachytherapy parameters between the two machines, i.e., source and applicators. Also, source activity may play a key role in the incidence of late rectal complications. For low-dose-rate brachytherapy, Labin *et al*. reported that the dose rate has no significant influence on local tumor control, but that significantly more rectal complications are observed in the higher dose rate group compared with the lower dose rate group [10]. In high dose rate Ir-RALS, Suzuki *et al*. [7] reported that the group with both a high BED (>100 Gy3) and a high activity (>2.4 cGy m^2^ h^−1^) showed significantly greater probability of late rectal bleeding compared with low BED and low activity groups.

BED can be converted into an equivalent dose of 2-Gy fractions (EQD2), and a rectal BED of 100 Gy3 is 60 Gy in EQD2. If the whole pelvic dose of EBRT was 30 Gy/15 fractions, (BED = 50), the rectal dose of RALS is restricted within about 20 Gy/5 fraction (BED = 47). The dose to the ICRU rectum point was not prospectively calculated for Group A and not reported in the present study, but this evaluation showed that the maximum permissible dose is ∼ 67% (20 Gy/30 Gy) of the Point A dose in Group A and 80% (20 Gy/25 Gy) of the Point A dose in Group B.

This study demonstrated almost the same local control rate in the two groups, regardless of the chemotherapy frequently used in Group B. Concurrent chemoradiotherapy (CCRT) has been shown to be superior to definitive radiotherapy (RT) alone in several randomized controlled trials, and is now the standard of care for locoregionally advanced uterine cervical cancer [11]. However, these trials were investigated with higher cumulative radiation dose schedules than those used in Japanese centers. In Japan, favorable local control results have been obtained with lower dose schedules in some retrospective series with RT alone [7, 12], however CCRT with these lower dose schedules have not been accepted practice in the USA and Europe given the lack of prospective data. Recently JGOG conducted a phase II study of CCRT with a low cumulative RT dose schedule and achieved comparable outcomes to those attained with global dose schedules, but with a lower incidence of late toxicity for locally advanced uterine cervical cancer [13]. We have not yet conducted prospective studies with RT alone and CCRT with lower dose schedules.

## CONCLUSION

In conclusion, this retrospective analysis suggests that low dose per fraction (5 Gy/fraction) with a tungsten shielded applicator and modified source step size is advantageous because it can achieve almost the same local control rate but with fewer rectal complications in high-dose rate Ir-RALS.
